# Genomic islands: tools of bacterial horizontal gene transfer and evolution

**DOI:** 10.1111/j.1574-6976.2008.00136.x

**Published:** 2008-10-30

**Authors:** Mario Juhas, Jan Roelof van der Meer, Muriel Gaillard, Rosalind M Harding, Derek W Hood, Derrick W Crook

**Affiliations:** 1 Clinical Microbiology and Infectious Diseases, Nuffield Department of Clinical Laboratory Sciences, University of OxfordOxford, UK; 2 Department of Fundamental Microbiology, University of LausanneLausanne, Switzerland; 3 Departments of Zoology and Statistics, University of OxfordOxford, UK; 4 Molecular Infectious Diseases Group, The Weatherall Institute of Molecular Medicine, University of OxfordOxford, UK

**Keywords:** horizontal gene transfer, genomic island, evolution, pathogenicity, biodegradation

## Abstract

Bacterial genomes evolve through mutations, rearrangements or horizontal gene transfer. Besides the core genes encoding essential metabolic functions, bacterial genomes also harbour a number of accessory genes acquired by horizontal gene transfer that might be beneficial under certain environmental conditions. The horizontal gene transfer contributes to the diversification and adaptation of microorganisms, thus having an impact on the genome plasticity. A significant part of the horizontal gene transfer is or has been facilitated by genomic islands (GEIs). GEIs are discrete DNA segments, some of which are mobile and others which are not, or are no longer mobile, which differ among closely related strains. A number of GEIs are capable of integration into the chromosome of the host, excision, and transfer to a new host by transformation, conjugation or transduction. GEIs play a crucial role in the evolution of a broad spectrum of bacteria as they are involved in the dissemination of variable genes, including antibiotic resistance and virulence genes leading to generation of hospital ‘superbugs’, as well as catabolic genes leading to formation of new metabolic pathways. Depending on the composition of gene modules, the same type of GEIs can promote survival of pathogenic as well as environmental bacteria.

## Introduction

Genomes of bacterial species can evolve through a variety of processes including mutations, rearrangements or horizontal gene transfer. Information gathered over the past few years from a rapidly increasing number of sequenced genomes has shown that besides the core genes encoding essential metabolic functions, bacterial genomes also harbour a variable number of accessory genes acquired by horizontal gene transfer that encode adaptative traits that might be beneficial for bacteria under certain growth or environmental conditions (Schmidt & Hensel, 2004).

Many of the accessory genes acquired by horizontal transfer form syntenic blocks recognized as genomic islands (GEIs). GEIs are typically recognized as discrete DNA segments between closely related strains, but it is currently thought that their formation contributes to the diversification and adaptation of microorganisms, thus having a significant impact on the genome plasticity and evolution, the dissemination of antibiotic resistance and virulence genes, and formation of catabolic pathways. More and more DNA elements are being detected by bacterial genome sequencing projects, and, at the same time, more information has become available on the life-style of GEIs. This review will summarize recent advances in our understanding of their distribution, evolution and mechanistic modes of behaviour. One of the emerging ideas is that GEIs comprise an overarching family of elements, including previously recognized mobile DNA elements such as integrative and conjugative elements (ICEs), conjugative transposons and some prophages. One GEI-type, a class of highly conserved ICEs occurring in *Beta*- and *Gammaproteobacteria* will be particularly highlighted.

## General features of GEIs

GEIs are in essence discrete DNA segments differing between closely related bacterial strains to which usually some past or present mobility is attributed. If we accept this definition it will comprise an overarching family of elements with different functional life-styles. The concept of pathogenicity islands (PAIs) was originally coined in the late 1980s by J. Hacker and colleagues, who investigated the genetic basis of virulence of uropathogenic isolates of *Escherichia coli* (UPEC) (Hacker *et al.*, 1990). PAIs found in their study were unstable chromosomal regions with variable virulence-associated characteristics and phenotypes (Groisman & Ochman, 1996). Nowadays, it is appreciated that GEIs represent a much broader and more diverse group of DNA elements than PAIs only, with a large variety of sizes and abundance in bacterial genomes (Dobrindt *et al.*, 2004). Different GEI families, some probably evolutionarily very ancient, have been recognized on the basis of predicted sequence and functional homologies (Burrus *et al.*, 2002; Juhas *et al.*, 2007b; Vernikos & Parkhill, 2008). The coding capacity of GEIs is not limited to pathogenicity functions, but can be very diverse, including such traits as symbiosis (Sullivan *et al.*, 2002), sucrose and aromatic compound metabolism (Gaillard *et al.*, 2006), mercury resistance and siderophore synthesis (Larbig *et al.*, 2002b). Bioinformatics studies have shown that GEIs tend to carry more ‘novel’ genes (i.e. those that do not have orthologues in other species) than the rest of the genome (Hsiao *et al.*, 2005). This suggests that GEIs have become strongly selected for adaptive and auxiliary functions. The fact that GEIs come in a large spectrum of varieties in terms of genetic organization and functionality makes it more difficult to provide an exact definition of a GEI. Here, we propose that the term GEI should be used for the overarching family of discrete ‘DNA elements’, which are part of a cell's chromosome and can drive or have driven strain differentiation.

**Fig. 1:**
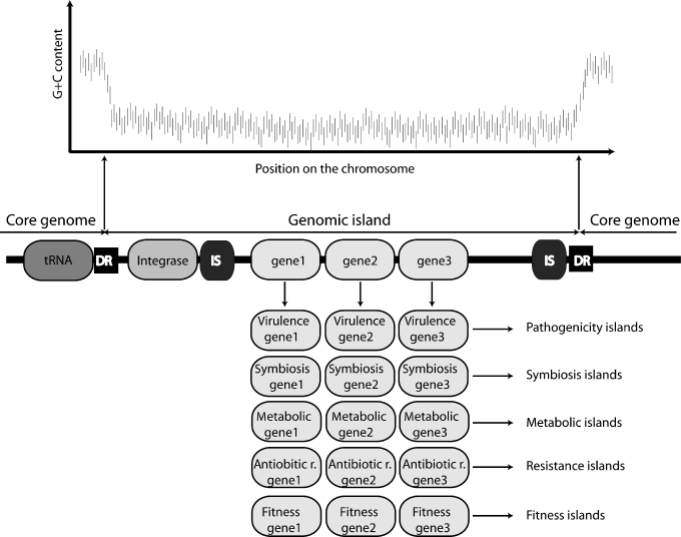
General features of GEIs. GEIs are relatively large segments of DNA whose nucleotide characteristics often differ from the rest of the chromosome. GEIs are often inserted at tRNA genes and flanked by DR. GEIs typically harbour genes encoding factors involved in genetic mobility, such as integrases, transposases and insertion sequences (IS). According to their gene content, GEIs can be described as pathogenicity, symbiosis, metabolic, fitness or resistance islands.

Most GEIs known to date share the following features (Fig. 1):

1. GEIs are relatively large segments of DNA, usually between 10 and 200 kb detected by comparisons among closely related strains. Discrete DNA regions detected by comparative genome sequencing with sizes smaller than 10 kb have been named genomic islets (Hacker & Kaper, 2000).
2. GEIs may be recognized by nucleotide statistics (e.g. GC content, cumulative GC skew, tetranucleotide frequencies or codon usage) that usually differ from the rest of the chromosome.
3. GEIs are often inserted at tRNA genes, in which case they might be ICEs.
4. GEIs are often flanked by 16–20-bp perfect or almost perfect direct repeats (DR). DRs usually arise by the site-specific integration of the GEIs into the target site and can act as recognition sequences for their enzymatic excision (Schmidt & Hensel, 2004).
5. GEIs often harbour functional or cryptic genes encoding integrases or factors related to plasmid conjugation systems or phages involved in GEI transfer.
6. GEIs often carry insertion elements or transposons, which may have been implicated in mobilizing genetic material onto or deleting DNA from the element (Buchrieser *et al.*, 1998; Gal-Mor & Finlay, 2006).
7. GEIs often carry genes offering a selective advantage for host bacteria. According to their gene content, GEIs are often described as pathogenicity, symbiosis, metabolic, fitness or resistance islands (Dobrindt *et al.*, 2004; Schmidt & Hensel, 2004).

## Evolutionary origins of GEIs

**Fig. 2:**
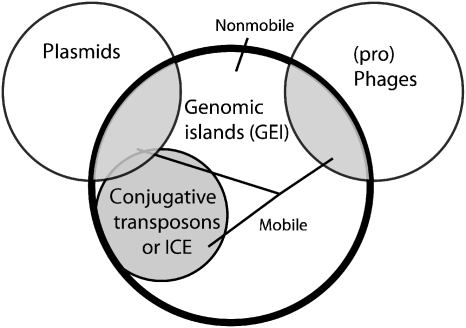
Variable types of GEIs. GEIs come in a large spectrum of varieties and encompass other categories of elements, such as conjugative transposons/ICEs, integrated plasmids, nonreplicative but excisable elements, and cryptic or damaged prophages. Grey-shaded areas point to self-mobile GEIs.

Although most of the GEIs known so far fit the above-described definition, a significant number of elements lack one or more of the hallmark indications. As many GEIs have only been put forward on the basis of data from sequencing projects, and not on experimental evidence of their mode of action, many elements may actually be in an evolutionary state of regression, as has been suggested earlier by Dobrindt *et al.* (2004). Sequence and phylogenetic comparisons show that GEIs tend to fall within structurally distinct genus-specific families, but looking across different genus boundaries leads to the emergence of universally distributed structural GEI components (Vernikos & Parkhill, 2008). This suggests that GEIs may have arisen multiple times independently during evolution, and can thus only be seen as a superfamily of elements on the basis of analogous core and structural features, rather than being phylogenetically related (Juhas *et al.*, 2007b; Vernikos & Parkhill, 2008). As the original definition of GEIs was put forward 10 years ago when only 12 complete bacterial genomes were available, it is plausible that other GEIs with novel and unusual features will be discovered with the increasing number of bacterial genomes being sequenced. In the more overarching view we propose here, GEI would encompass other categories of elements, such as ICE/conjugative transposons [which were proposed to be one functionally similar group of ICEs (Burrus *et al.*, 2002; Burrus & Waldor, 2004)], integrated plasmids, nonreplicative but excisable elements [nonreplicating *Bacteroides* unit (NBU) from *Bacteroides* (Shoemaker *et al.*, 2000)], and perhaps even cryptic or damaged prophages (Fig. 2).

Work over the last few years has suggested that GEIs have had multiple parallel evolutionary origins, as some of them clearly contain phage and others conjugative plasmid-related genes. In addition, it has become clear that mobile elements form by a combination of functional modules, which makes it more difficult to categorize them (Toussaint & Merlin, 2002; Frost *et al.*, 2005; Leplae *et al.*, 2006). For example, as it is common to find multiple prophages in various stages of functionality in a bacterial chromosome, it is plausible that some have cointegrated with the chromosome and slowly acquired other genes of nonphage origin (Dobrindt *et al.*, 2004; Klockgether *et al.*, 2004). Indeed, for a number of GEIs, the similarities between GEI-encoded and known plasmid transfer systems are so striking that one can rightfully assume that the plasmid transfer genes formed the origin for the progenitor hybrid GEI element. This is the case, for example, for the *Mesorhizobium loti* R7A symbiosis island (Sullivan *et al.*, 2002; Ramsay *et al.*, 2006), the CTn*4371* biphenyl transposon of *Ralstonia oxalatica* (Toussaint *et al.*, 2003) and the SXT element of *Vibrio cholerae* (Beaber *et al.*, 2002). However, this need not necessarily be the case for all GEIs. Recent experiments with a family of ICEs with deep evolutionary relationships suggested that some of them may have evolutionarily very ancient self-transfer modes for which no current plasmid relatives are known. This conclusion was drawn from observations for three members of this family, namely ICE*Hin1056* from *Haemophilus influenzae*, pKLC102 from *Pseudomonas aeruginosa* and ICE*clc* from *Pseudomonas* sp. strain B13. These three elements are fully functional elements, capable of integration into the chromosome of the host, excision and self-transfer by conjugation to a new host and reintegration (Dimopoulou *et al.*, 2002; van der Meer & Sentchilo, 2003; Gaillard *et al.*, 2006; Klockgether *et al.*, 2007; Juhas *et al.*, 2007a). As will be outlined further below, their self-transfer system forms a distinct type IV secretion system (T4SS), which is only very distantly related to other known plasmid-encoded T4SSs.

## Integration, development and excision of GEIs

As not all GEIs have the same components, it is difficult to speak of one unifying mode of GEI functioning or life-style (i.e. those functions necessary for maintenance, excision, transfer or integration). Interestingly, a large number of GEIs for which self-mobility has been demonstrated, can excise from their chromosomal location, encode the full capacity for horizontal self-transfer to another cell, and reintegrate into the target site in the new host's chromosome. GEIs that exhibit simultaneously all these features and self-transfer by conjugation are part of an increasingly well-defined group of elements that have been named ICEs (Burrus & Waldor, 2004). ICEs also include conjugative transposons, a terminology that had been used mostly for elements that originated in gram-positive bacteria, and, according to some authors, should be reserved for elements, which can target multiple different integration sites (Burrus *et al.*, 2002). As some GEIs do not self-transfer by conjugation but by phage packaging, release and infection, they cannot be called ICEs.

**Fig. 3:**
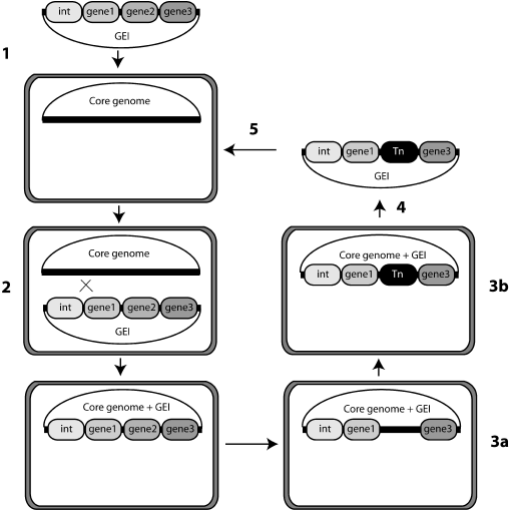
Integration, development and excision of GEIs. The schematic life-style of mobile GEI consists of the following steps: (1) acquisition by horizontal gene transfer; (2) integration into the host's chromosome by site-specific recombination; (3) development of the GEI by genetic rearrangements, gene loss (a) or gene acquisition (b); (4) excision from the chromosome; (5) transfer to another recipient.

The schematic life-style of mobile GEI would thus consist of the following steps (Fig. 3):

1. Acquisition of the GEI by a host through horizontal gene transfer.
2. Integration of the GEI into the host chromosome by site-specific recombination.
3. Development of the GEI by genetic rearrangements, gene loss or acquisition of other mobile genetic elements.
4. Excision of the GEI from the chromosome.
5. Transfer of the GEI to another recipient.

As stated above, many GEIs may be in a state of evolutionary regression, with the result that one or more functionalities are missing. A number of active GEIs have been studied concerning various levels of life-style, coding capacity or regulation of transfer, and the results have contributed to knowledge about the general mechanistic modes of GEIs. For reasons only partially understood, GEIs are often inserted in the 3′-end of tRNA genes (Ravatn *et al.*, 1998a; Larbig *et al.*, 2002b; Williams, 2002). Insertion is catalyzed by site-specific phage-like recombinases called integrases, which are usually encoded by the GEI itself. Integrases are not strictly conserved among all GEIs, and different tRNA genes can be targeted (Williams, 2002). Several GEI-encoded integrases relate to the lambda, P4 or XerD families (van der Meer *et al.*, 2001; Burrus *et al.*, 2002; Mohd-Zain *et al.*, 2004). The integrases are also implicated in the excision of GEIs, a process that may be assisted by an excisionase (Burrus & Waldor, 2003; Lesic *et al.*, 2004; Ramsay *et al.*, 2006). The *int* gene encoding integrase is often situated at one extremity of the island and adjacent to the tRNA gene in the integrated GEI form. Following excision, both GEI ends close up to form a single copy of the recombination site (*attP*) (Ravatn *et al.*, 1998b; Doublet *et al.*, 2005; Qiu *et al.*, 2006; Ramsay *et al.*, 2006). Excision is mostly nonreplicative and a single copy of the recombination site is again formed on the chromosome (*attB*) (Burrus & Waldor, 2003; Ubeda *et al.*, 2007). GEI can reintegrate from the excised form back into the *attB* site of the same host or, after transfer to a new cell, in a suitable *attB* site in a new cell. Integration is the result of a site-specific recombination between a 15- and 20-bp motif within the 3′ extremity of the tRNA gene (Ravatn *et al.*, 1998a; Williams, 2002). Except in a few cases (Klockgether *et al.*, 2004; Ubeda *et al.*, 2007), excised GEIs do not seem to replicate independently from the host's chromosome but rely instead on reintegration or horizontal transfer to a new host and reintegration to proliferate. Intermediate forms may also exist, as was demonstrated in a recent study on staphylococcal pathogenicity islands (SaPIs), which are phage-type GEIs. SaPIs, which have intimate relationships with certain temperate phages involving phage-induced excision, replication and packaging, were also proven to be capable of an existence in a self-replicating plasmid-like state (Ubeda *et al.*, 2007, 2008). A large body of knowledge exists on integration and excision processes of conjugative transposons in, for example, *Bacteroides*, the details of which, however, lie outside the scope of this review.

## Transfer of GEIs between bacteria

As suggested above, a wide variety of GEIs are intimately connected to phages and conjugative plasmids through their evolutionary origins. As a consequence, besides transformation, their transfer often proceeds via conjugation and transduction (Jain *et al.*, 2002; Chen *et al.*, 2005). GEIs do not necessarily encode the whole process of self-transfer, and several cases are known in which GEIs can be packaged by another coresiding lysogenic phage or mobilized by a plasmid or ICE conjugative system (Shoemaker *et al.*, 2000).

Transformation has been observed in a number of gram-positive or gram-negative, pathogenic or environmental bacteria. Naturally transformable bacteria such as *Acinetobacter* sp. ADP1, *Bacillus subtilis, Streptococcus pneumoniae, Neisseria gonorrhoeae, Pseudomonas stutzeri, Ralstonia solanacearum* and *H. influenzae* take up free DNA from their surrounding environment (Smith *et al.*, 1995; Hamoen *et al.*, 2001; Barbe *et al.*, 2004; Meier & Wackernagel, 2005; Fall *et al.*, 2007). Part of the foreign DNA acquired by natural transformation is degraded, but part could be incorporated into the host's genome, thus contributing to the evolution of bacterial species. Naturally transformable bacteria need to acquire a physiological state called ‘competence’ through expression of a number of genes encoding DNA uptake and processing systems before transformation. Uptake systems of naturally transformable bacteria are often composed of components resembling subunits of the type IV pili and type II secretion systems (Chen *et al.*, 2005). Several bacteria, including *N. gonorrhoeae* and *H. influenzae*, have a strong bias to take up species-specific DNA for transformation. Efficient species-specific DNA uptake in *N. gonorrhoeae* and *H. influenzae* requires the presence of an *c*. 10-nucleotide-long DNA uptake sequence, which is found at a high frequency (*c*. 1400 copies) in the respective genomes (Goodman & Scocca, 1988; Smith *et al.*, 1995). Recently, a novel T4SS has been identified in *N. gonorrhoeae* that secretes chromosomal DNA in the surrounding environment in a noncontact-dependent manner (Hamilton *et al.*, 2005). This T4SS is localized in the large, horizontally acquired gonococcal genetic island (GGI) present in the chromosome of *N. gonorrhoeae*; thus by facilitating chromosomal DNA secretion, this GEI also encodes the mechanism of its own dissemination. Interestingly, the SOS response to antibiotic stress-induced DNA damage has been shown to induce genetic transformability of bacteria and hence to promote horizontal dissemination of antibiotic resistance genes (Beaber *et al.*, 2004; Prudhomme *et al.*, 2006).

Conjugation is the process of DNA transfer from donor to recipient through a specialized apparatus that consists of a cell-envelope spanning translocation channel joined to a tube-like structure known as a pilus in gram-negative bacteria or to the surface-associated adhesins in gram-positive bacteria (Chen *et al.*, 2005). Conjugation systems constitute part of a large and versatile family of T4SS-dependent transport systems (Chen *et al.*, 2005; Christie *et al.*, 2005). T4SSs are usually encoded by multiple genes organized into a single operon. Based on the organization of genetic determinants, shared homologies and evolutionary relationships, T4SSs have been classified into several types (Juhas *et al.*, 2008). Type F and P T4SSs, often referred to as type IVA systems, resemble the archetypal VirB/VirD4 system of *Agrobacterium tumefaciens* and are considered to be the paradigm of type IV secretion. Type I T4SSs, also referred to as type IVB systems, resembling the archetypal Dot/Icm system are found in two intracellular bacterial pathogens, *Legionella pneumophila* and *Coxiella burnetii* (Segal *et al.*, 2005). The most recently described T4SSs that are evolutionarily distant from all previously described GI T4SSs, play a key role in the horizontal transfer of a wide variety of GEIs derived from a broad spectrum of bacteria, including *Haemophilus* spp., *Pseudomonas* spp., *Erwinia carotovora, Salmonella enterica* serovar Typhi, *L. pneumophila* and others (Juhas *et al.*, 2007a, b, 2008). This lineage of T4SSs has been named GI-type to emphasize the fact that it was found to be associated only with certain GEIs (Juhas *et al.*, 2007a).

Transduction is the process of DNA transfer from one bacterium to another via bacterial viruses, bacteriophages. Many bacteriophages are able to transfer bacterial genes, including GEIs, as passengers in their genomes. One good example is the SaPI family of *Staphylococcus aureus* islands (Maiques *et al.*, 2007). Members of this family were shown to be induced to excise and replicate by certain resident temperate phages that also play a role in their packaging into a small phage-like particles (Ubeda *et al.*, 2003, 2007; Maiques *et al.*, 2007) that are transferred from donor to recipient cells at frequencies commensurate with the plaque-forming titer of the phage (Ruzin *et al.*, 2001). Interestingly, SaPIbov2, a member of the SaPI family of GEIs, has been shown to be induced to replicate by different staphylococcal phages, encapsidated and transferred to a variety of recipient bacteria, including different *Staphylococcus* strains (Maiques *et al.*, 2007). Certain genomic regions of staphylococci resemble slightly deteriorated prophages that could be mobilized by other phages. GEI transfer may mechanistically resemble certain aspects indicative of an existence of phage- and plasmid-like ancestors: the transfer of such islands may include self-replicating plasmid-like states (Ubeda *et al.*, 2007). Several other examples of GEIs from a wide variety of bacterial species have been reported recently to be transferred by bacteriophages, including *Yersinia* high-pathogenicity island (HPI) of *Yersinia pseudotuberculosis* (Lesic *et al.*, 2004) or GEIs of the marine cyanobacterium *Prochlorococcus* (Coleman *et al.*, 2006).

Active GEI transfer has been described in a number of cases, although for the great majority of potential GEIs detected by genome sequencing projects, no information is as yet available on their transfer capacities. For example, conjugative transfer of ICE*Hin1056*, calculated as a number of transconjugants divided by a number of recipients, proceeds at a frequency of around 10^−1^–10^−2^ between two *H. influenzae* strains (Juhas *et al.*, 2007b). ICE*clc* of *Pseudomonas* sp. strain B13, a distant member of the same ICE*Hin1056* subfamily, is self-transferable at similar frequencies to *P. putida, Cupriviadus necator* or *P. aeruginosa* (Gaillard *et al.*, 2006). Recently, another member of this ICE*Hin1056* subfamily of GEIs, *P. aeruginosa* pathogenicity island-1 (PAPI-1) was shown to transfer from donor strain into *P. aeruginosa* recipient strains that do not harbour this island naturally. Furthermore, PAPI-1 has been demonstrated to exist in an extrachromosomal circular form and to reintegrate into the host genome following excision from the chromosome (Qiu *et al.*, 2006). Finally, also the GEI pKLC102 of *P. aeruginosa* was shown to be highly mobile and capable of transferring at the frequencies similar to ICE*clc* or ICE*Hin1056* (Klockgether *et al.*, 2007). Interestingly, however, no excision or transfer has been demonstrated for other members of this subfamily of GEIs such as PAGI-2 and PAGI-3 (Klockgether *et al.*, 2007).

Recent research indicates that the host background has a strong influence on the transferability of GEIs. The T4SS transfer module was found to be one of the most conserved parts of the ICE*Hin1056* subfamily of GEIs (Juhas *et al.*, 2007b). However, the analysis of the conjugation transfer frequencies showed that these GEIs were transferred between closely related haemophili strains with significantly different frequencies. To test whether these differences were due to the respective host strain background or to the gene content of the island, the GEIs of haemophili were transferred from the same donor into the same recipient strain. In this setting, the conjugal transfer frequencies were almost constant, which is indicative of the different host strains introducing variations in the conjugal efficiency in the initial experiment (Juhas *et al.*, 2007b). Interestingly, also transfer frequencies of ICE*clc* were largely dependent on the type of donor cell, even within closely related strains of *P. aeruginosa* (Gaillard *et al.*, 2008). These studies have clearly shown that host background has a tremendous impact on the transferability of GEIs.

## Regulation of GEIs and adaptive behaviour

There is still little information available on the regulation of or environmental conditions influencing GEI transfer. In fact, for most GEIs it was initially assumed that transfer would be ‘spontaneous’ or ‘constitutive’. However, it has been suggested that in a number of cases, tightly regulated events underlay the onset of GEI self-mobilization (Jain *et al.*, 2003). This is not so surprising, especially given the high level of control on the life-style of temperate bacteriophages. A better understanding of the conditions for self-transfer, the factors augmenting or decreasing transfer rates, and the (self-)regulation of the onset of the transfer process is of great importance, both for our appreciation of the impact of horizontal gene transfer on the evolution of microorganisms and for the practical purpose of judging the potential distribution of transgenes or antibiotic resistance genes in natural microbial populations (Nielsen & Townsend, 2004). Evidence from the few GEI models studied so far suggests indeed quite the opposite of ‘spontaneous’ behaviour: a variety of regulatory modes and signals, which determine GEI self-transfer.

One of the most striking regulatory modes for GEI behaviour was revealed from studies on the tetracycline determinants in *Bacteroides*. Conjugative transfer of the ICEs CTn*DOT* and CTn*ERL* from *Bacteroides* is stimulated up to 10 000-fold when cells are grown in the presence of tetracycline. This effect was found to be the result of an induction of two regulatory genes, *rteA* and *rteB*, both of which stimulate transcription of a third factor *rteC*, which influences excision of the element (Cheng *et al.*, 2001; Whittle *et al.*, 2002). From the work on ICE*clc* of *Pseudomonas* sp. strain B13, we know that ICE*clc* transfer is strongly enhanced in the stationary phase in a bistable fashion (e.g. only *c*. 5% of all cells engage in transfer). Transfer of ICE*clc* correlates to an increase of expression from the *intB13* integrase gene, which is stimulated by the product of the gene *inrR* (Sentchilo *et al.*, 2003a). Upon excision and formation of a circular intermediate, a strong promoter – otherwise located at the other end of ICE*clc* facing outwards – is placed in front of the *intB13* gene favouring the reintegration process (Sentchilo *et al.*, 2003a). Transfer of the 108-kb GEI called PAPI-1, which was discovered in *P. aeruginosa* strain PA14 and is similar to the element pKLC102, proceeds via excision, formation of an intermediate circular form and reintegration into either of the two *tRNA*^*Lys*^ genes in *P. aeruginosa* (PA4541 and PA0976) (Qiu *et al.*, 2006). The authors of this work could demonstrate that a *soj* gene encoded by PAPI-1 itself was required for the maintenance of the element, both in integrated and in circular form (Qiu *et al.*, 2006). Their hypothesis was that Soj protects the circular form of PAPI-1 either directly from degradation or indirectly by promoting the integration of the circular form back into the chromosome. The major evidence for this was the finding that *soj* is expressed by the circular form at early stationary phase. The Soj protein is related to the ParA family of proteins, which are responsible for correct segregation of low-copy plasmids during cell division.

Transfer regulation proceeds differently in the SXT element of *V. cholerae*. Excision of SXT is favoured by an excisionase Xis (Burrus & Waldor, 2003), which, however, also inhibits its integration. In SXT, *xis* and *int* are convergent genes that do not appear to be coregulated. SXT transfer is strongly enhanced under stress conditions and is dependent on the SOS response (Beaber *et al.*, 2004). Interestingly, the *V. cholerae* SOS response is eluded in particular by two antibiotics, ciprofloxacin and trimethoprim, for which the SXT element encodes resistance determinants. The mechanism is thought to proceed as follows: in the presence of an SOS stimulus, the SXT-encoded repressor SetR is cleaved, resulting in the expression of two SXT-encoded genes *setC* and *setD*, which are activators for the *int* and *tra* genes of the element (Burrus & Waldor, 2003). Also excision of ICE*Bs1*, a mobile element found in the genome of *B. subtilis*, is stimulated by global DNA damage in addition to an intercellular peptide signaling. This behaviour was found to be dependent on the factor ImmR, which regulates expression of a number of ICE*Bs1* genes and is responsible for immunity to superinfection (Auchtung *et al.*, 2007).

Excision of the ICE*MISymR7A* symbiosis island of *M. loti* strain R7A is also stimulated by a novel recombination directionality factor (RDF) called RdfS, which is encoded by the gene *msi109* (Ramsay *et al.*, 2006). Transfer of the ICE*MISymR7A* also requires a putative relaxase, RlxS. The genes *rdfS* and *rlxS* are part of the same cluster of which two other genes are homologues to the conjugative protein TraF (Ramsay *et al.*, 2006). Similar to the *clc* element, also the excised form of ICE*MISymR7A* was more abundant in stationary than exponential phase of *M. loti*, and experimental evidence suggested that this excision was under quorum-sensing control (Ramsay *et al.*, 2006).

Another example for excisionase requirement is the integrase of the HPI, which cannot alone promote efficiently the excision of HPI. In this case, it was demonstrated that a factor called Hef RDF, which is encoded by HPI, is required for excision (Lesic *et al.*, 2004). Although the level of *hef* expression severely affected the rate of HPI excision, it had little or no effect on *int* transcription, and the authors concluded that Hef could not act as transcriptional regulator. Another RDF called Rox (for regulator of excision) was shown to stimulate excision of the *Shigella* resistance locus PAI in *Shigella flexneri* (Luck *et al.*, 2004). Similarly to Hef, this Rox protein is not an activator of *int* transcription, although it showed 66% sequence similarity to AlpA from the phage CP4-57. AlpA is a transcription factor for the *int* gene of the phage CP4-57 in *E. coli* K-12, regulating the excision of the prophage from the bacterial chromosome (Trempy *et al.*, 1994). The examples described above suggest that GEI transfer can be a highly regulated process with a variety of developed regulatory modes.

As discussed below, GEIs play an important role in bacterial genome evolution in general and in adaptation to changing conditions, in clinical, industrial or natural environments. The outcome of these adaptations is obvious from the development of antibiotic resistance, pathogenicity or catabolic functions. Thus, it would be extremely interesting to find specific features, which make GEIs so successful in self-transfer, host-entry or establishment in a host. Are GEIs considered parasites by a new host? Do GEIs have the means to trick a host and avoid its defence systems? Are all GEIs alike in this respect or do exceptions exist? Relatively little information is available concerning these questions and the nature of GEI–host interactions in general.

Most of the information on the host–DNA invader interactions comes from conjugative plasmids and phages. In general, the frequency of successful DNA exchange between bacteria belonging to different genera will depend on many factors: the degree of homology between the transferred DNA and the bacterial host, the metabolic compatibility, adaptations to their abiotic environment, gene expression systems, gene-transfer mechanisms, the mismatch repair and restriction endonuclease systems. For example, the transfer efficiency of the broad-host range IncP-1 plasmid RK2, as measured by the number of transfer events per donor present, was dramatically influenced by the nature of the donor–recipient combination: between *E. coli* strains or from *E. coli* to *P. putida*, RK2 transfer was much less frequent than between *P. putida* strains. This was attributed to species-specific differences in RK2 gene expression (Bingle & Thomas, 2001). The recipient cell can also limit the entry or establishment of the incoming DNA by surface exclusion, a process by which a barrier seems to be created by cells that already carry the genes for a closely related transfer apparatus (Frost *et al.*, 1994). Horizontally acquired DNA that confers a selective advantage to the host obviously has the potential to spread further among suitable recipients within a bacterial population under the appropriate selective conditions (Thomas & Nielsen, 2005).

Upon successful transfer, the newly incoming DNA must still be maintained to ensure its long-term survival in the new host. If there is a clear selective advantage conferred by the acquired DNA for the host, and no major fitness cost under nonselective conditions, it is less likely that the horizontally acquired DNA will be lost. Incoming DNA on a plasmid must be able to replicate independently but synchronously the host's chromosomal replication and cell division. This process is usually guaranteed by a plasmid-specific system, such as killing daughter cells without partitioned plasmids. In the absence of appropriate replication, plasmids may still ‘survive’ by recombining into the host's chromosome, which is promoted by the presence of suitable sequences for homologous recombination. On the other hand, GEIs depend on site-specific reintegration into an appropriate chromosomal target site, in the absence of which the element is unlikely to be maintained. However, once integrated, a GEI will be automatically maintained by chromosomal replication. Loss can occur when the GEI excises, as there seems to be a strong advantage for growth of cells without GEIs. Such a scenario was demonstrated recently in a study on the UPEC *E. coli* isolate 536, which contains five PAIs, some of which can become deleted during chronic infection. The authors could show that the integrase of one of the PAIs could actually mediate the excision of another PAI. This suggests the existence of unidirectional cross-talk between integrases of different PAIs (Hochhut *et al.*, 2006). Similar cross-talk phenomena had been detailed previously by work on the *Bacteroides* element NBU1, which can be coexcised and mobilized by the conjugative transposon CTnERL (Shoemaker *et al.*, 2000).

Although it is generally stated that GEIs give a selective advantage to the host cell (Dobrindt *et al.*, 2004), this has not been extensively experimentally tested yet. In fact, results from conjugative plasmids would suggest the opposite, namely that cells which acquire a conjugative plasmid, go through a period of fitness loss (Dahlberg & Chao, 2003). This fitness loss can be caused by particular functions carried on the plasmid which are detrimental upon expression in the new host (e.g. regulatory proteins interfering with the global gene expression network), or by proteins expressed from plasmid which have a direct phenotypic effect (e.g. antibiotic resistance) (Nguyen *et al.*, 1989). Bacterial populations having received a newly incoming plasmid were shown to adapt to their previous fitness by spontaneous mutations which reduce or repress the plasmid specific effects (Dahlberg & Chao, 2003). Plasmids with both very low to unmeasurable cost, and very large cost to the host were detected and their ability to persist in a bacterial population was shown to be influenced by the host strain background (De Gelder *et al.*, 2007; Schluter *et al.*, 2007). Very recently, it was demonstrated that ICE*clc* had only a very minor effect on the fitness of host *P. aeruginosa* strains (Gaillard *et al.*, 2008). Although so far only a single case of this has been found, it might point to this type of ICE/GEI having specific mechanisms to reduce fitness cost in the host (Gaillard *et al.*, 2008).

There is increasing evidence that GEI expression is globally influenced by the host in which it resides. Thus, GEIs usually constitute part of global regulatory networks and genes on the particular GEI can be regulated by regulators present on the same GEI, by regulators harboured by other GEIs or by regulators encoded by the host bacterium. Similarly, GEI-borne regulators often play a role in the regulation of genes on the bacterial chromosome.

Regulators frequently contributing to regulation of GEIs comprise the two-component response regulator family, AraC family, alternative sigma factors and histone-like proteins. Paradigmal regulatory networks involving intensive cross-talk between GEI-borne regulators and gene components of the host genome include *Salmonella* pathogenicity islands (SPI-1 and SPI-2) of *S. enterica, Vibrio* pathogenicity island (VPI) of *V. cholerae* and the locus of enterocyte effacement (LEE) of *E. coli* and have been reviewed elsewhere (Hacker & Kaper, 2000; Schmidt & Hensel, 2004).

Recent studies into the regulatory role of the histone-like nucleoid-structuring protein (H-NS) have shed a new light into the regulation of some bacterial GEIs. H-NS is a pleiotropic regulator that modulates gene expression of gram-negative bacteria in response to environmental stimuli, such as temperature and osmolarity (Hommais *et al.*, 2001). H-NS represents the bacterial functional equivalent of histones, plays an important role in a local supercoiling of DNA, and has higher affinity for curved DNA (Navarre *et al.*, 2006). Work from several laboratories exploiting recent DNA microarray technology has shown that H-NS plays a key role in the selective silencing of horizontally acquired genes (Lucchini *et al.*, 2006; Navarre *et al.*, 2006). Expression of >400 genes was shown to be upregulated in the *Salmonella hns* mutant, out of which more than 90% were acquired by the horizontal gene transfer (Navarre *et al.*, 2006). The GC content of most of the H-NS repressed genes was lower than the average GC content of the host *Salmonella* genome, thus leading to the conclusion that H-NS can selectively silence horizontally acquired genes by targeting sequences with proportionally high AT content (Navarre *et al.*, 2006). Recently, conserved sequence motifs have been identified that represent the high-affinity DNA-binding sites for H-NS (Lang *et al.*, 2007). In accordance with previously published studies, these motifs occur in AT-rich regions of DNA both within operons and in genes harboured by the pathogenicity-associated GEIs (Lang *et al.*, 2007). As described above, the differences in the nucleotide statistics are among the characteristic features of GEIs, thus targeting sequences with different GC content by H-NS represents an elegant and efficient mechanism of regulation of newly acquired GEIs.

## Contribution of GEIs to horizontal gene transfer and bacterial evolution

It is widely recognized that horizontal gene transfer facilitated by GEIs has played a crucial role in the evolution of bacterial species. This is attributed not only to simple acquisition and loss of GEI-borne genes, but also to the possibility of GEIs transferring parts of a host's chromosomal DNA. Upon excision from the host genome, GEIs can play a role in the transfer of parts of the host chromosome into the recipient bacteria (Hochhut *et al.*, 2000). Furthermore, the presence of a wide variety of secretion systems on many GEIs suggests that these can be used not only for the transfer of GEIs and the GEI-encoded products, but also for the transfer of the host's chromosomal DNA. One good example is the secretion of chromosomal DNA in the gonococcus via the GGI-encoded T4SS (Hamilton *et al.*, 2005). Chromosomal DNA secreted via the GGI-encoded type T4SS may be subsequently taken up by natural transformation, thus facilitating recombination that contributes to antigenic variation and the spread of antibiotic resistance (Hamilton *et al.*, 2005). GEIs could undergo a recombination with the host's chromosome, with a significant impact on the evolution of the host bacterium, as GEI-borne genes often encode important clinical or fitness traits. Whether encoding genes are involved in pathogenicity or biodegradation, GEIs can facilitate evolution by ‘quantum leaps’ as their acquisition or loss can rapidly and dramatically alter the life-style of a bacterium (Groisman & Ochman, 1996; Ochman *et al.*, 2000; van der Meer & Sentchilo, 2003; Vernikos *et al.*, 2007). It should be noted that many of the genes found in GEIs are novel and of unknown function, with no detectable homologues in other species, but nevertheless they might have a role and confer selective advantage to the host organism (Hsiao *et al.*, 2005).

## Contribution of GEIs to evolution of pathogenic bacteria

Antibiotic resistance represents one of the most frequent and well-studied traits associated with GEIs. The emergence and dissemination of antibiotic resistance is a serious threat to public health as it puts the successful treatment of infectious diseases in increasing doubt. This change in bacterial populations from near universal susceptibility to increasing antibiotic resistance worldwide in a few decades is illustrative of the remarkable capacity for bacterial adaptation, albeit a man-made threat. Many aspects of the accumulation of antibiotic resistance are poorly understood. One prominent aspect is how such resistance has disseminated globally so rapidly.

Investigations into the emergence of antibiotic resistance in *H. influenzae* suggest that it is an illustrative model for how resistance genes have become associated with a GEI. Sequence analysis of an *H. influenzae* antibiotic-resistant GEI, ICE*Hin1056*, revealed that this island belonged to much larger family (Mohd-Zain *et al.*, 2004; Dimopoulou *et al.*, 2007). Diverged GEIs of this family coevolve independently with a wide range of *Proteobacteria*, including *Haemophilus* spp., *Pseudomonas* spp., *Yersinia enterocolitica, S. enterica* serovar Typhi and *Ralstonia metallidurans* (Mohd-Zain *et al.*, 2004). A recent study investigating GEIs of haemophili has shown that the ICE*Hin1056* subfamily of ICE/GEIs is diverse and has not recently emerged in haemophili (Juhas *et al.*, 2007b). However, the low-sequence diversity and distinctive GC content of the Tn*3*s found in the GEIs of haemophili suggest more recent acquisition of the transposon-borne antibiotic resistance genes. Distribution between core and accessory genes (transposons) of the GEIs of haemophili is remarkably similar to the host *H. influenzae*‘supragenome’ and conforms to the distributed genome hypothesis (Juhas *et al.*, 2007b). This hypothesis proposes that the full complement of genes available to a pathogenic bacterial species exists in a supragenome pool, which is not contained by any particular strain (Hogg *et al.*, 2007). The modular structure of the core genes of the GEIs of haemophili suggests that they are functionally constrained and, acting in concert, they play a major role in the successful propagation and survival of these GEIs within their hosts' ‘supragenomes’. Their importance is overtly manifested by the rapid dissemination of antibiotic resistance worldwide among *H. influenzae* and *Haemophilus parainfluenzae* strains (Juhas *et al.*, 2007b). The recent acquisition of antibiotic resistance genes and their rapid global spread over the past 30–40 years is an example of how GEIs contribute to bacterial diversification and adaptation.

*Staphylococcus aureus* is a potentially pathogenic bacterium that plays a role in a broad spectrum of diseases and is a major cause of hospital-acquired infections worldwide. Methicillin-resistant *S. aureus* (MRSA) often referred to as the hospital ‘superbug’ in the press, represents one of the most serious threats to public health due to its resistance to a wide variety of antibiotics. This antibiotic resistance is facilitated mostly by genes located on a GEI-designated staphylococcal cassette chromosome *mec* (SCC*mec*) (Katayama *et al.*, 2000). The five SCC*mec* subtypes identified so far range in size from 20 to 70 kb and confer resistance to methicillin, kanamycin, tobramycin, bleomycin, penicillins, heavy metals, tetracycline, macrolide, lincosamide and streptogramin (Ito *et al.*, 2001; Deurenberg *et al.*, 2007). GEI SCC*mec* is interesting in a sense that it does not contain phage-related and *tra* genes or transposases and is transferred between bacteria with the help of two site-specific recombinases that catalyze its chromosomal excision and reintegration (Ito *et al.*, 2004; Noto & Archer, 2006). The origin of GEI SCC*mec* remains to be elucidated; however, it is hypothesized that it could originate from other staphylococci, namely *Staphylococcus sciuri* or *Staphylococcus epidermidis*. This is suggested by the high-amino acid sequence similarities of the methicillin resistance gene *mecA* products of *S. sciuri* and *S. aureus* as well as by an experiment where the *S. sciuri mecA* gene induced methicillin resistance in a formerly methicillin-sensitive *S. aureus* strain (Wu *et al.*, 2001). Furthermore, *S. aureus mecA* was identical to that identified in an *S. epidermidis* isolate from the same individual, thus suggesting that MRSA strain has arisen *in vivo* by horizontal transfer of *mecA* between two staphylococcal species (Wielders *et al.*, 2001; Deurenberg *et al.*, 2007). While one theory hypothesizes that all MRSA clones have a common ancestor (Kreiswirth *et al.*, 1993), another theory suggests that SCC*mec* was introduced several times into different *S. aureus* lineages (Musser & Kapur, 1992; Fitzgerald *et al.*, 2001; Enright *et al.*, 2002; Qi *et al.*, 2005).

*Enterococcus faecalis* is also one of the leading agents of nosocomial infections of surgical sites, the urinary tract and bloodstream (Richards *et al.*, 2000). Most of the virulent strains of *E. faecalis* harbour a 150-kb GEI consisting of 129 ORFs, including those encoding a wide variety of toxins, cytolysin and the surface proteins Esp and aggregation substance (Shankar *et al.*, 2002). Recently, the horizontal transfer of the *E. faecalis* GEI has been demonstrated (Coburn *et al.*, 2007). This has many implications for the evolution and diversity of *E. faecalis*, as it suggests a mechanism for the conversion of commensal *E. faecalis* strains to virulent ones by acquisition of the GEI-encoded virulence traits (Coburn *et al.*, 2007).

Results from recent studies have shown that GEIs also played a key role in the evolution of pathogenic human and mammalian *Mycobacterium* spp. (Gutierrez *et al.*, 2005; Becq *et al.*, 2007). Speciation events in ancestral *Mycobacterium* spp. that were initially environmental bacteria occurred only relatively recently, about a million years ago, and are linked to the invasion of the genome by foreign DNA (Gutierrez *et al.*, 2005; Rosas-Magallanes *et al.*, 2006; Becq *et al.*, 2007). Several *Mycobacterium tuberculosis* GEIs harbour genes that have been identified previously as virulence genes in other bacteria (Pethe *et al.*, 2004; Stewart *et al.*, 2005; Becq *et al.*, 2007). A good example illustrating the role played by the horizontally acquired GEIs in the evolution of *Mycobacterium* spp. pathogenic to mammals, is GEI Rv0986-8, as the Rv0986-8-borne genes are required for the binding of *M. tuberculosis* to eukaryotic cells and *in vitro* trafficking (Pethe *et al.*, 2004; Becq *et al.*, 2007; Rosas-Magallanes *et al.*, 2007).

Siderophore-mediated iron uptake is important for pathogenic as well as environmental bacteria. HPI is a 36–43-kb GEI that encodes the siderophore yersiniabactin-mediated iron uptake system (Lesic *et al.*, 2004). This GEI was first found in *Yersinia* spp. but has been subsequently identified in a broad spectrum of *Enterobacteriaceae*. The presence of highly homologous HPI-borne ORFs among different species suggests recent acquisition of the HPI by these bacteria; however, the exact mechanism mediating the horizontal transfer of these islands awaits further investigation (Lesic & Carniel, 2005).

Many bacterial species exploit specialized secretion systems to transfer macromolecules across bacterial membranes. GEIs of a wide variety of bacterial pathogens encode type III secretion systems (T3SS) and T4SS, which by transfer of proteins or nucleoprotein complexes directly mediate pathogenicity and horizontal gene transfer. *Salmonella enterica* serovar Typhi is a gram-negative facultative intracellular pathogen that is a causative agent of gastroenteritis and typhoid fever (Shea *et al.*, 1996). The divergence of *Salmonella* and *E. coli* from their common ancestor occurred *c*. 100–140 million years ago and these bacterial species have each acquired and lost more than 3 Mb of novel DNA since their divergence (Vernikos *et al.*, 2007). The first stage of *Salmonella* infection, characterized by the colonization and invasion of intestinal epithelial cells, is usually followed by the replication within host's macrophages. Two T3SSs harboured by two different GEIs, SPI-1 and SPI-2, play a crucial role in the pathogenesis of *S. enterica*. T3SS of SPI-1 is important for the penetration of intestinal epithelium, and T3SS of SPI-2 has been hypothesized to be required solely after bacterium has gained access to the host's macrophages. Recent findings with a mouse model of typhoid indicate that this process is even more complicated, as SPI-2 was shown to be expressed also during early stages of pathogenesis before penetrating the intestine. This suggests that in addition to other functions, SPI-2 may be also involved in preparing *Salmonella* to successfully resist the harsh antimicrobial environment within macrophages (Brown *et al.*, 2005). T3SSs harboured by *Salmonella* islands SPI-1 and SPI-2 are only two of many examples of GEI-borne T3SSs enhancing the pathogenicity of their host bacteria. Besides T3SSs, GEIs can also encode T4SSs. Various T4SSs associated with GEIs include for instance the GI-like family of T4SSs described above. The T4SSs are unique among other bacterial secretion systems due to their ability to transfer both proteins and nucleoprotein complexes. They can deliver bacterial effector proteins to host cells, thus contributing directly to pathogenicity. Furthermore, they can mediate horizontal gene transfer, thus facilitating the evolution of pathogens through dissemination of virulence genes (Juhas *et al.*, 2008).

Adhesion, either interbacterial or to specific receptors of host cells, represents another important pathogenicity trait that is often associated with GEIs. VPI encodes the toxin-coregulated pilus (TCP) that represents the major intestinal adherence factor of *V. cholerae*, the causative agent of cholera (Karaolis *et al.*, 1999). TCP is a type IV pilus that mediates interbacterial adherence, resulting in formation of microcolonies, as well as secretion of a soluble colonization factor, TcpF, crucial for successful colonization of host (Kirn *et al.*, 2003). Importance of the VPI-encoded TCP is overtly manifested by the inability of the TCP-deficient mutants of *V. cholerae* to colonize and cause disease in both humans and mice (Tripathi & Taylor, 2007).

Other GEI-encoded adherence factors include P fimbriae and S fimbriae of uropathogenic *E. coli* (UPEC) and intimin of enteropathogenic and enterohaemorrhagic *E. coli* (EPEC and EHEC, respectively) (reviewed elsewhere, see Hacker & Kaper, 2000). Both EPEC and EHEC infections are the leading cause of infantile diarrhoea. Multiple separate acquisitions of the LEE GEI and of the EPEC adherence factor plasmid (EAF) seem to be responsible for the evolution of the EPEC pathotype within *E. coli* (Lacher *et al.*, 2007). Furthermore, microarray and whole genome PCR scanning analyses have shown that LEE GEI constitutes one of the most conserved parts of different EHEC strains. This study suggests that independent infections of similar but distinct bacteriophages carrying virulence determinants, including LEE GEI, are deeply involved in the evolution of EHEC strains belonging to different *E. coli* lineages (Ogura *et al.*, 2007). Horizontal transfer of GEIs was also shown to play a major role in the evolution of a number of other bacterial pathogens, including LIPI (listeria pathogenicity island) of *Listeria monocytogenes* (Vazquez-Boland *et al.*, 2001), SHI-0, SHI-1, SHI-2 of *S. flexneri* (Nie *et al.*, 2006), BfPAI of *Bacteroides fragilis* (Franco, 2004) and cag of *Helicobacter pylori* (Gressmann *et al.*, 2005).

## Contribution of GEIs to evolution of environmental bacteria and making the link to pathogenicity

Interestingly, although GEIs have been increasingly associated with the rise and distribution of virulence functions or antibiotic resistance genes, at the same time they appear to be implicated in a multitude of other adaptive traits, which collectively could be considered of ‘environmental relevance’. For example, a number of ICE/GEIs have been identified in *Beta-* and *Gammaproteobacteria*, which carry gene clusters for the degradation of chlorinated and nitroaromatic compounds (Gaillard *et al.*, 2006) or biphenyls (Toussaint *et al.*, 2003). Even dehalogenases in *Dehalococcoides ethenogenes* have been associated with integrated DNA elements, but whether these are mobile or not remains to be determined (Seshadri *et al.*, 2005).

**Fig. 4:**
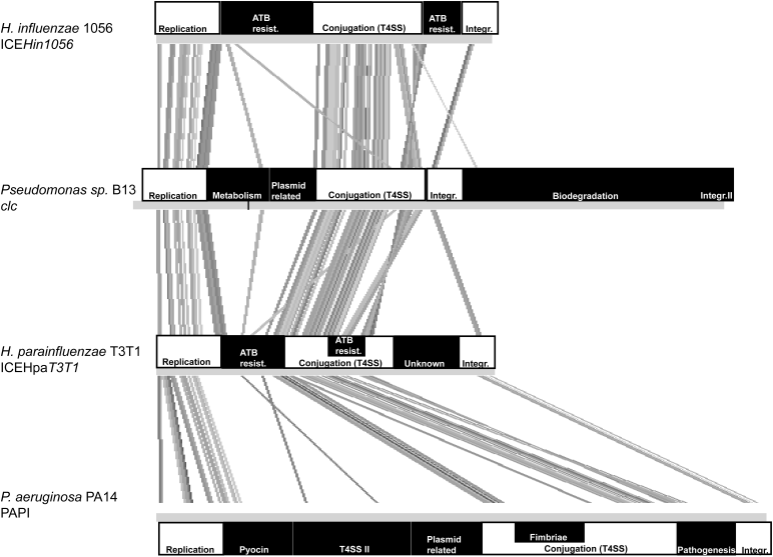
Various functions encoded by GEIs of the same family. Modified Artemis Comparison Tool view of four GEIs of the same family: ICE*Hin1056, clc*, ICE*HpaT3T1* and PAPI. Homologous sequences (minimum cut-off=50) are indicated by grey lines joining regions of the four GEIs. Gene modules homologous in all shown GEIs are represented by white boxes, and modules specific for individual GEIs by black boxes. The figure shows that depending on the composition of gene modules, members of the same family of GEIs can promote survival of pathogenic (ICE*Hin1056*, ICE*HpaT3T1*, PAPI of *Haemophilus influenzae, Haemophilus parainfluenzae* and *Pseudomonas aeruginosa*, respectively) as well as environmental (*clc* of *Pseudomonas* sp. B13) bacteria. ATB resist., antibiotics resistance; T4SS, type IV secretion system; Integr., integration.

One of these ICE/GEIs, the highly mobile 103-kb ICE*clc* of *Pseudomonas* sp. strain B13, often used as a model for the behaviour of catabolic GEIs (Sentchilo *et al.*, 2003a, b), is a good example of how GEIs can contribute to the adaptation of environmental strains to use polluting compounds as new carbon sources. Various classical experiments have used *Pseudomonas* sp. strain B13 to develop new metabolic pathways in a single step, such as for chlorobiphenyl or chlorobenzene degradation (Reineke, 1998). The recently revealed molecular basis for this metabolic ‘complementation’ of recipient bacteria also showed that ICE*clc* is in a continuing state of further adaptation. First of all, ICE*clc* carries a 9.5-kb *amn* gene cluster for aminophenol degradation with an aberrant nucleotide usage, suggesting its past acquisition by a precursor element. Then, the genome of the environmental bacterium *Burkholderia xenovorans* LB400 contains an element almost 100% identical to ICE*clc*, except for additional genes allowing degradation of *o-*halobenzoates (Chain *et al.*, 2006; Gaillard *et al.*, 2006). Finally, in the groundwater isolate *Ralstonia* sp. strain JS705 an element similar to ICE*clc* was detected with a 10-kb insertion of another catabolic pathway gene cassette, necessary for chlorobenzene degradation (Muller *et al.*, 2003). This development model for ICE*clc* of acquisition (and loss) of gene modules became even more striking when it was discovered that a region of about 40% of the catabolic ICE*clc* elements is highly similar to a number of other ICE/GEIs (Larbig *et al.*, 2002b; Mohd-Zain *et al.*, 2004; Gaillard *et al.*, 2006) (Fig. 4). This region was therefore suspected to contain the information for self-transfer, which was more recently confirmed by the analysis of the T4SS genes of the ICE*Hin1056* from *H. influenzae* that showed significant homology to counterpart ICE*clc* genes (Juhas *et al.*, 2007a). The core region of this group of elements was found in a variety of other bacteria (Fig. 4), thus showing that this evolutionarily ancient element has been very successful in transferring into a large host-range (Mohd-Zain *et al.*, 2004; Juhas *et al.*, 2007a). More recently, GEI fragments similar to ICE*clc* were also detected in *Xylella fastidiosa, Xanthomonas campestris, Rubrivivax gelatinosus, Azoarcus, Cupriviadus* and the arsenic-oxidizing bacterium *Herminiimonas arsenicoxydans* (Gaillard *et al.*, 2006; Muller *et al.*, 2007).

Particularly interesting was the close similarity between ICE*clc* and a large widely distributed group of GEIs of *P. aeruginosa*, including PAGI-2 and PAGI-3 (Larbig *et al.*, 2002a, b; Klockgether *et al.*, 2007). PAGI-2 and PAGI-3 were initially characterized from one clinical and one environmental *P. aeruginosa* strain (Larbig *et al.*, 2002b), but were found to be very common in a large screening of clinical and environmental isolates of *P. aeruginosa*, in addition to the even more promiscuous element pKLC102/PAPI-1 (Wolfgang *et al.*, 2003; He *et al.*, 2004; Qiu *et al.*, 2006; Klockgether *et al.*, 2007). The genetic load, in addition to the core region, was very different between ICE*clc*, PAGI-2 and PAGI-3, demonstrating that these GEIs can easily accommodate and distribute regions of 60–70 kb of highly dissimilar DNA. The fact that this genetic load is found mostly between the integrase gene (near one end of the element) and the core region suggests that insertions here are selectively neutral and do not compromise the functionality of the GEI. A majority of accessory genes were also found to be located in this position in the closely related *Haemophilus* spp. ICE*Hin1056* subfamily of GEIs (Juhas *et al.*, 2007b). This GEI-type is therefore an extreme example of the near continuum between characteristics judged as pathogenicity (ICE*Hin1056* of *H. influenzae*, or PAGI-2 and PAGI-3 of *P. aeruginosa*) and environmentally (ICE*clc* of *Pseudomonas* sp. strain B13) related (Fig. 4).

Various other recent examples of whole genome comparisons show that GEIs have been implicated in adaptation to a wide range of different life-styles and carry a large variety of functions. For example, the 49.6-kb GEI AGI-3 of *E. coli* EXPEC strain BEN2908, which is integrated in the *selC* tRNA locus, encodes various sugar transporters and sugar metabolic functions (Chouikha *et al.*, 2006). *Burkholderia cenocepacia* isolates often harbour an unstable 31.7-kb GEI which provides its host bacterium with the ability to synthesize *N*-acyl homoserine lactones and fatty acids and also encodes further amino acid transporters and metabolic genes (Baldwin *et al.*, 2004). Furthermore, *Corynebacterium efficiens* carries four GEIs, of 70, 55, 40 and 32 kb, one of which (CEGI4) has the most characteristics of a fully functional ICE. Apart from an integrase, all other ORFs encode unknown proteins (Zhang & Zhang, 2005). Recent comparison of several *P. aeruginosa* isolates has revealed the presence of multiple novel GEIs. One of those, the GEI RGP29 in *P. aeruginosa* PA2192, is a 224-kb element containing within its sequence another discernible GEI, called the *dit* island. The *dit* island encodes a full metabolic pathway for abietane diterpenoids (Mathee *et al.*, 2008). In addition, the RGP5 element of *P. aeruginosa* PA14 encodes various transporters and genes known to be involved in iron metabolism (Mathee *et al.*, 2008). *Magnetospirillum gryphiswaldense* contains a 130-kb unstable region of probable ancient GEI origin, but now filled with insertion elements (42 copies) that cause frequent recombinations and deletions of parts of this genomic region. Interestingly, this region encodes many genes involved in magnetosome biomineralization (Ullrich *et al.*, 2005). Suspected ancient GEIs with deteriorated integrases for which no transfer or excision could be demonstrated were detected in the anaerobic bacterium *Geobacter sulfurreducens* (Butler *et al.*, 2007). One such region has a size of 300 kb and contained many genes implicated in anaerobic metabolism of benzoate, phenol, *p*-cresol and 4-hydroxybenzoate. Free-living water and soil-borne bacteria with no specific pathogenicity characteristics, but which are thought to provide reservoirs for more pathogenic bacteria, have now also been found to harbour GEIs. One of those is *Arcobacter butzleri*, an Epsilonproteobacterium related to *H. pylori* (Miller *et al.*, 2007). Three potential GEIs have been found in this bacterium, the largest of which (ABG11) had a size of 26.9 kb and was integrated in *tRNA*^*Leu*^ with an integrase gene nearby. The island encodes 29 genes, none of which, however, have known orthologues in other bacterial genomes. More recent genome comparisons of multiple *X. fastidiosa* and *X. campestris* strains have again reinforced the importance of GEIs in strain differentiation and provision of potential virulence functions (da Silva *et al.*, 2007; He *et al.*, 2007). For example, *Xylella* strains from citrus fruits carried one or more copies of the GI1 island, which encodes fimbrillin synthesis, haemolysin production and lipopolysaccharide synthesis (da Silva *et al.*, 2007). Various rearrangements were observed in the largest (100 kb) island of *X. campestris* named XVR13 integrated in *tRNA*^*Gly*^ (He *et al.*, 2007).

## Concluding remarks

Work over a number of years has shown the importance that GEIs have and have had in promoting horizontal gene transfer and distributing a wide range of adaptive functions for the host bacterium. GEIs seem to do the same job as many self-transferable plasmids. Conceptually, GEIs may have a number of advantages over a plasmid, one of the most notable being that GEIs are integrated in the host's chromosome. Thus, unlike replicating plasmid molecules, GEIs do not need to continuously ensure coordinated replication, partitioning or specific maintenance, and because there is often only a single copy of the GEI present per genome, its replication ‘cost’ may not be as heavy a burden to the host cell (Gaillard *et al.*, 2008). However, under certain conditions, those GEIs that are functionally mobile can undergo excision, self-transfer and reintegration into a new host. More interestingly, several findings, including tetracycline-induced transfer of the conjugative transposon CTn*Dot* of *Bacteroides* (Cheng *et al.*, 2000), DNA damage and SOS stress response-induced transfer of the SXT element of *V. cholerae* (Beaber *et al.*, 2004), and 3-chlorobenzoate-augmented transfer of the *clc* element of *Pseudomonas* sp. strain B13 (Sentchilo *et al.*, 2003b), indicate a much more regulated train of events for the transfer of GEIs that is fine-tuned in response to environmental signals. Thus, the question arises whether unwittingly, by changing the conditions for bacteria in hospitals (antibiotic stress) and environment (pollution), we are generating selective conditions which promote the success of self-transferable and responsive GEIs. As shown on multiple examples in this review, GEIs play a crucial role in the evolution of a broad spectrum of pathogenic or environmental bacteria. Furthermore, several lines of evidence suggest the existence of evolutionary ancient GEIs spread over versatile groups of otherwise unrelated bacterial species. Besides a conserved set of core genes required for maintenance, these evolutionary ancient GEIs also harbour a variable number of other gene modules whose composition is strongly dependent on the life-style of the particular host bacterial species, which can be either pathogenic or environmental (Fig. 4). The important contribution of many research groups around the world, has led to GEI-facilitated horizontal gene transfer being one of the most rapidly evolving fields of microbiology research.

## Statement

Re-use of this article is permitted in accordance with the Creative Commons Deed, Attribution 2.5, which does not permit commercial exploitation.
